# Trans-kingdom fungal pathogens infecting both plants and humans, and the problem of azole fungicide resistance

**DOI:** 10.3389/fmicb.2024.1354757

**Published:** 2024-02-12

**Authors:** Alexandra Pintye, Renáta Bacsó, Gábor M. Kovács

**Affiliations:** ^1^Centre for Agricultural Research, Plant Protection Institute, HUN-REN, Budapest, Hungary; ^2^Department of Plant Anatomy, Institute of Biology, Eötvös Loránd University, Budapest, Hungary

**Keywords:** emerging fungal pathogens, *Aspergillus*, trans-kingdom pathogens, cross-resistance, resistance markers, DMI fungicides, *Rhizopus*, *Fusarium*

## Abstract

Azole antifungals are abundantly used in the environment and play an important role in managing fungal diseases in clinics. Due to the widespread use, azole resistance is an emerging global problem for all applications in several fungal species, including trans-kingdom pathogens, capable of infecting plants and humans. Azoles used in agriculture and clinics share the mode of action and facilitating cross-resistance development. The extensive use of azoles in the environment, e.g., for plant protection and wood preservation, contributes to the spread of resistant populations and challenges using these antifungals in medical treatments. The target of azoles is the cytochrome p450 lanosterol 14-α demethylase encoded by the *CYP51* (called also as *ERG11* in the case of yeasts) gene. Resistance mechanisms involve mainly the mutations in the coding region in the *CYP51* gene, resulting in the inadequate binding of azoles to the encoded Cyp51 protein, or mutations in the promoter region causing overexpression of the protein. The World Health Organization (WHO) has issued the first fungal priority pathogens list (FPPL) to raise awareness of the risk of fungal infections and the increasingly rapid spread of antifungal resistance. Here, we review the main issues about the azole antifungal resistance of trans-kingdom pathogenic fungi with the ability to cause serious human infections and included in the WHO FPPL. Methods for the identification of these species and detection of resistance are summarized, highlighting the importance of these issues to apply the proper treatment.

## Introduction

Fungicides are the first line of treatments to control fungal diseases of plants, humans, and animals. Among the different types of antifungals, azoles are the most widely used and this is the only group applied both in medicine and in the environment (Snelders et al., 2012). Fungal infections affect hundreds of millions of people worldwide and cause more than 1.7 million deaths annually (Mota Fernandes et al., 2021). For the most devistating diseases, like invasive aspergillosis, the mortality rate can be as high as 90% (Mota Fernandes et al., 2021) and azole antifungals are the primary treatment options (Meis et al., 2016). Demethylation inhibitor (DMI) fungicides (including azoles) are broadly used to control postharvest diseases, plant pathogenic fungi in agricultural fields, and preservation purposes. Around 2 million tons of agricultural azole fungicides were sold in 2020, and more than two-thirds of them were sold in Europe and Asia, occupying about 16% of the global fungicide volume market (Jørgensen and Heick, 2021). In wood preservation, fungicides are used to increase the durability of wood and inhibit the growth of decay fungi, and about 18% of the fungicides used for wood preservation are azoles (Jørgensen and Heick, 2021).

**Figure 1:**
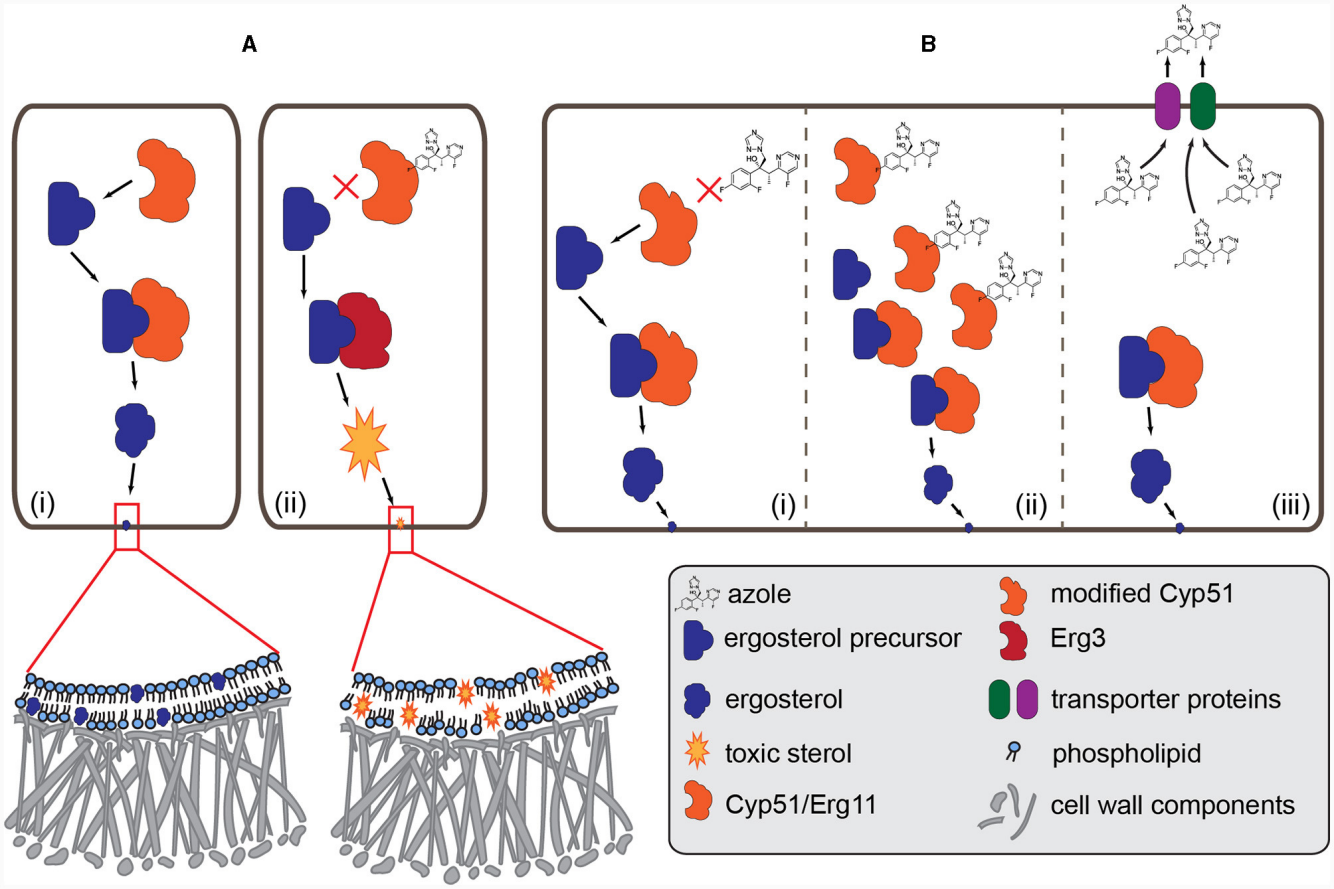
Mode of action of azole fungicides and the major resistance mechanisms. **(A)** (i): normal biosynthesis of ergosterol. The Cyp51/Erg11 (cytochrome P450 lanosterol 14-α demethylase) enzyme demethylases the precursor and then the matured ergosterol is integrated into the membrane. (ii): azole antifungals (e.g., voriconazole) bind to Cyp51 and block the production of ergosterol. Intermediate toxic sterol produced by the enzyme Erg3 accumulates, altering the fungal membrane integrity. **(B)** (i): amino acid changes in the Cyp51 enzyme, caused by point mutations and promoter insertions in the *CYP51* gene, may change the structure of the protein, resulting in resistance to azoles. (ii): overexpression of the Cyp51 enzyme due to the changes in the promoter region or increased copy number of the *CYP51* coding gene. (iii): the amount of azoles decreases in the fungal cell due to the overexpression of the efflux pump (MFS and ABC transporters) genes.

Azoles inhibit the ergosterol biosynthesis pathway by blocking the cytochrome P450 lanosterol 14-α demethylase encoded by the *CYP51* gene (Becher and Wirsel, 2012), which has three paralogs (see below) in Pezizomycotina (Fungi, Ascomycota; Van Rhijn et al., 2021; Celia-Sanchez et al., 2022; Figure 1). Blocking the Cyp51 enzymes leads to the alteration of the fungal membrane integrity and the accumulation of deleterious sterols (Becher and Wirsel, 2012). In agriculture propiconazole, difenoconazole and tebuconazole are the most commonly used azoles (Ishii et al., 2021), while fluconazole (FLC), itraconazole (ITC), posaconazole (POS), and voriconazole (VRC) are the main antifungals during the treatment of humans infected by fungi—besides of amphotericin B (AMB) belonging to the polyene class, and echinocandins (Gisi, 2014). Although the chemical structure of those azoles are different, cross-resistance develops quickly because of the identical mode of action of the agricultural and clinical azoles (Gisi, 2014). For a detailed figure about the molecular structures of azoles and other antifungal compounds see Snelders et al. (2012).

Several species (e.g., *Fusarium solani*) have so-called intrinsic or primary resistance to one or more members of azole group (or any other antifungals), which is independent of previous exposure to these chemicals (Al-Hatmi et al., 2016). The secondary or acquired resistance develops when a pathogen has come into contact with antifungals, which can happen in the environment or during medical treatment (Gisi, 2014).

The widespread application contributes to the spread of azole-resistant genotypes of different species, including opportunistic pathogens like *Aspergillus fumigatus* (Verweij et al., 2013) and so-called trans-kingdom fungal pathogens like the *Fusarium oxysporum* species complex. The emergence of azole-resistant strains gained more attention during the SARS-CoV-2 pandemic because viral pneumonia increases patients' susceptibility to bacterial and fungal superinfections (Koehler et al., 2021). The viral infection leads to immune dysfunction, and the secondary infection by fungicide-resistant species like *A. fumigatus* (Armstrong-James et al., 2020) or *Rhizopus oryzae* (Rao et al., 2021) became an additional contributing factor to mortality (Koehler et al., 2021).

Trans-kingdom fungal pathogens, capable of infecting plants and mammals, belong mainly to Ascomycota and Mucoromycota (Gauthier and Keller, 2013) and a few to Basidiomycota (Hu et al., 2021). These particular pathogens usually affect people with impaired immunity, e.g., those who have undergone organ transplantation (Gauthier and Keller, 2013). For multihost and crossover pathogens, *Fusarium oxysporum* is an outstanding example. *Fusarium* species are serious emerging pathogens of both humans and plants with a broad spectrum of antifungal resistance (Al-Hatmi et al., 2016; World Health Organization, 2022), underlining that the widespread application of azoles in agriculture poses a severe threat to the use of antifungals in the clinics and contributes to the loss of efficiency of these chemicals. The invasive fungal infection by a tomato pathogenic isolate of *F*. *oxysporum* and death of the immunodepressed mice were proven in pathogenicity tests, and it was also shown that the same virulence factors play functionally distinct roles in plant and animal pathogenesis (Ortoneda et al., 2004).

The World Health Organization (WHO) has just issued the first fungal priority pathogens list (WHO FPPL) to raise awareness of the risk of fungal infections and the increasingly rapid spread of antifungal resistance, thus strengthening the need for a global response to those problems (World Health Organization, 2022). Three priority groups (critical, high, and medium) of fungal pathogens were assigned based on multicriteria decision analysis. Prioritization criteria included for example, the average case fatality rate, level of acquired or intrinsic resistance to antifungal treatment and availability of diagnostics. The fungal species included in the final priority list cause mainly systemic invasive infections and are associated with high mortality and or morbidity (World Health Organization, 2022).

**Figure 2:**
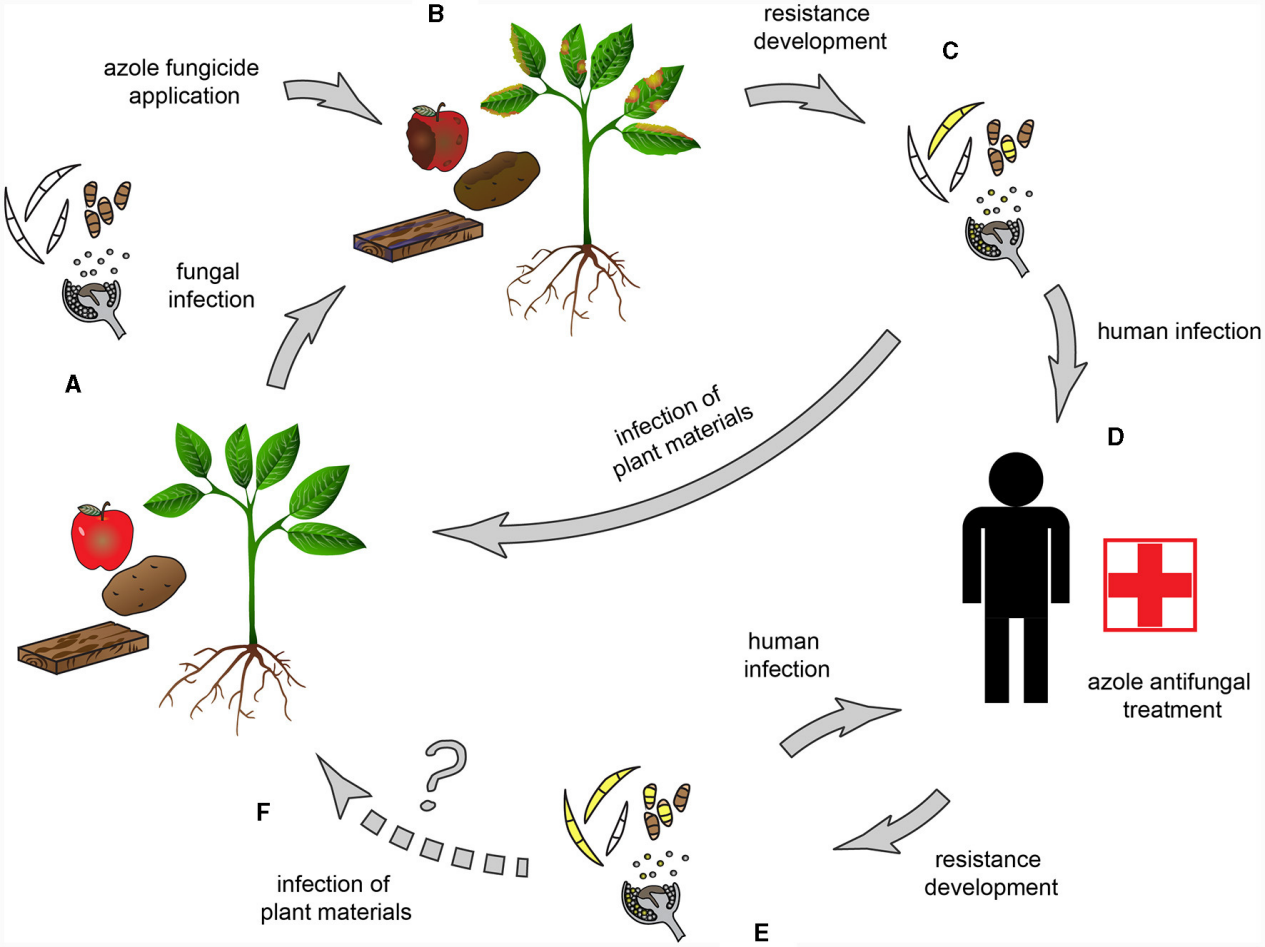
Possible routes of resistance development in trans-kingdom pathogens. **(A)** Trans-kingdom fungal pathogens, capable of infecting both plants and humans, are the causal agents of e.g., blight diseases, foliar necrotic spots, postharvest fruit rot, and bluing of wood. **(B)** Azoles, including DMI fungicides, are broadly used to control phytopathogenic fungi. **(C)** Resistance develops and/or spreads when pathogens come into contact with fungicides. The widespread fungicide application contributes to the spread of azole-resistant genotypes. **(D)** Possible human infection by a trans-kingdom pathogen **(E)** Cross-resistance in sensitive fungi develops due to the identical mode of action of the agricultural and clinical azoles. **(F)** An open question is whether the transmission to plants could happen by trans-kingdom pathogens originating from human infections.

This review summarizes information about *CYP51* paralogs and the key aspects of the azole resistance of those species included in the WHO FPPL (World Health Organization, 2022) which have agricultural connections and are or could be trans-kingdom pathogens. The resistance mechanisms and methods for detecting the fungal species and resistance are discussed, and the possible connection with plant pathology and agriculture as a source of problems for human health is highlighted (Figure 2). Only critical and high priority groups are discussed since the medium group, albeit there are fungi with a saprobic part of their lifecycle, as far we know, does not include species with a strong, direct connection with agriculture.

## CYP51 paralogs

Until these days, five major gene family groups of *CYP51* have been reported in Fungi: *CYP51, CYP51A, CYP51B, CYP51C*, and *CYP51D* (Van Rhijn et al., 2021; Celia-Sanchez et al., 2022). Basidiomycota, Saccharomycotina, Taphrinomycotina and early-diverging fungi (Blastocladiomycota, Chytridiomycota, Monoblepharidomycota, Zoopagomycota, and Mucormycota) have only *CYP51* while the filamentous Ascomycota taxa, fungi in the Pezizomycotina subphylum, have either 1 or 2 or 3 copies of 4 *CYP51* paralogs (Van Rhijn et al., 2021; Celia-Sanchez et al., 2022). It was reported earlier (Caramalho et al., 2017) that three species from Mucoromycota (*Rhizopus arrhizus, R. microsporus*, and *Mucor circinelloides*) have paralogs (*CYP51* F1 and *CYP51* F5), however, in *R*. *miscrosporus* Celia-Sanchez et al. (2022) considered those paralogs as variants of *CYP51* (*CYP51.1* and *CYP51.2*).

According to Celia-Sanchez et al. (2022) all filamentous Ascomycota have *CYP51B* paralog, and only 50% have a *CYP51A* paralog. A gene duplication event could be the origin of the *CYP51A* and *CYP51B* paralogs followed by multiple losses of *CYP51A* (Hawkins et al., 2014). The presence of *CYP51C* was described from roughly 10 genera or species and all are pathogens. The sequences of *CYP51A, CYP51B*, and *CYP51C* are ~60% identical, and the Cyp51 and Cyp51B proteins have 88% of shared amino acids in motifs (Celia-Sanchez et al., 2022). Both the Cyp51A and Cyp51B proteins are localized in the perinuclear and peripheral endoplasmic reticulum (ER) in the apical and subapical hyphal compartments and occasionally along the hyphal septa and apical tips (Roundtree et al., 2020).

Functional diversification was described between paralogs in several fungal species (Fan et al., 2013). In the case of the plant pathogen *Fusarium graminearum CYP51C* is essential in the colonization of the wheat ears and does not encode an enzyme with sterol demethylase activity. *CYP51A* and *CYP51B* both encode sterol 14a–demethylase; however the latter is essential for ascospore formation, and the former plays an important role in azole resistance (Fan et al., 2013). It was shown that point mutations in *CYP51*, considered by the authors as C paralog, can contribute to the reduced azole susceptibility in clinical and plant pathogenic *Aspergillus flavus* isolates (Hermida-Alava et al., 2021). In other species, like the human pathogen *A. fumigatus*, the function of *CYP51A* and *CYP51B* can be complementary or even synergistic (Chen et al., 2020; Roundtree et al., 2020; Handelman et al., 2021). Research using deletion mutants of *CYP51A* and *CYP51B* showed that the paralogs can exhibit different susceptibility to azoles and differentially bind these fungicides (Chen et al., 2020; Roundtree et al., 2020).

The *CYP51D* paralog was recently described by Van Rhijn et al. (2021) from azole-resistant *Penicillium* and *Talaromyces* isolates.

## The critical priority group from the WHO FPPL

This group includes fungal species that have the highest perceived public health importance, namely: *A. fumigatus, Cryptococcus neoformans, Candida auris*, and *C*. *albicans*.

These fungi are not trans-kingdom pathogens, however the former two species strongly connect with agriculture, wood industry, and soils (Bastos et al., 2021). The fungi in the “critical group” (World Health Organization, 2022) will be briefly reviewed here because the environmental origin and azole resistance of those pathogens were discussed in detail by Bastos et al. (2021).

*Aspergillus fumigatus* is one of the most well-known and devastating causal agents of human mycoses, and the most common species involved in invasive aspergillosis (Verweij et al., 2020). It is also a saprotrophic, soil-born fungus, generally found on decaying plant material, e.g., in compost and flower bulb waste (Schoustra et al., 2019), air and soil samples of sawmills (Jeanvoine et al., 2017), and agricultural fields (Alvarez-Moreno et al., 2019). The environmental origin of the azole resistance of *A. fumigatus* has been repeatedly and undoubtedly shown (Verweij et al., 2013; Buil et al., 2019). This fungus is not a plant pathogen; therefore “agronomically used DMI fungicides are not directly targeted *A. fumigatus*, resistance selection is an unintended side effect” (Verweij et al., 2020).

*Cryptococcus neoformans* is a basidiomycetous yeast, besides other, medically less relevant *Cryptococcus* species, causes infections called cryptococcosis, including life-threatening meningoencephalitis (Idnurm et al., 2005). The occurrence of this soil-born fungus is often associated with animal droppings (Chen et al., 2021). *Cryptococcus neoformans* is not a plant pathogen, but it can be frequently isolated from decaying wood material, like hollows of carob trees and eucalyptus (Cogliati et al., 2016). On the other hand, as a common soil fungi it can be found in agricultural soils, too (Takahashi et al., 2020). Cross-resistance was observed between agricultural azole fungicides and clinical azoles several times in the case of environmental and clinical isolates (Bastos et al., 2018; Chen et al., 2021). Moreover, non-azole fungicides, like pyraclostrobin, can induce resistance to clinical azoles through overexpression of efflux pump genes (Bastos et al., 2019).

*Candida* species are responsible for many diseases, ranging from superficial mycosis to invasive candidiasis (Pappas et al., 2018). It has been recently shown that human pathogenic species, like *C. albicans*, survive in the environment for a prolonged time and can also be plant-associated, e.g., with oak trees (Bensasson et al., 2019; Opulente et al., 2019). It has been suggested as a possibility that the azole resistance in *Candida* species has environmental origins and it can be a result of the cross-resistance to agricultural fungicides (Morio, 2020; Castelo-Branco et al., 2022).

## The high priority group from the WHO FPPL

### *Rhizopus arrhizus* (syn. *R. oryzae*)

The order Mucorales is included in the high priority group of the WHO FPPL, of which *R. arrhizus*, an opportunistic human pathogen, is the most frequent causative agent of mucormycosis, and the leading cause of rhino–orbital–cerebral infections (Walther et al., 2019; Osaigbovo et al., 2023). This fungus causes ~70% of human mucormycoses (Roden et al., 2005) and mortality can be as high as 68–80% (Rabie and Althaqafi, 2012; Jeong et al., 2019; Osaigbovo et al., 2023). Usually the most common sites of infection are the skin, lungs, gastrointestinal tract, and paranasal sinus. The latter is the most dangerous because of the rapidly progressing rhinocerebral infections (Wickes, 2013). *Rhizopus arrhizus* cause deadly infections mainly in immuno-compromised individuals and people suffering from hyperglycemia, diabetes mellitus in ketoacidosis, other forms of metabolic acidosis, and other underlying conditions like treatment with corticosteroids, organ or bone marrow transplantation, neutropenia, traumatic disruption of the skin, and deferoxamine therapy in patients receiving hemodialysis (Ribes et al., 2000; Ibrahim et al., 2012; Jeong et al., 2019). In the case of SARS-CoV 2 and mucormycosis co-infection, due to the inappropriate establishment of the diagnosis and therapy, the mortality rate can be extremely high, more than 90%, as it was in India during the SARS-CoV2 pandemic in 2021 (Dam et al., 2023).

This fungus is characterized by rapid growth rates, aseptate hyphae, and wide distribution often found in soil, dung, and rotting vegetation. Due to its saprobic lifestyle, *R. arrhizus* produces a wide variety of commercially valuable metabolites (e.g., enzymes, alcohols, and organic acids) and is one of the leading producers of Asian fermented foods, like tempeh (Wickes, 2013). *Rhizopus arrhizus* (syn. *R. oryzae*) is also known as a plant pathogen, e.g., the causal agent of root rot (Haddoudi et al., 2021), and postharvest diseases of many species (Khokhar et al., 2019).

#### Pathogenesis and virulence factors

Infection by *R. arrhizus* (and other mucoralean species) can happen mainly through inhalation of the sporangiospores and traumatic disruption of the skin (e.g., burned wounds and usage of non-sterile adhesive tapes; Ibrahim et al., 2012; Choudhary et al., 2021). In the case of rhino- orbital-cerebral infection, penetration into the central nervous system occurs either by hematogenous spread or by direct cranial penetration from the paranasal sinuses (Chikley et al., 2019). Before the hyphal invasion of the brain tissue and the fatal outcome, a vascular occlusion leads to cerebral infarction and hemorrhagic necrosis in the central nervous system mucormycosis (Chikley et al., 2019).

During infection by *R. arrhizus* several virulence factors, which enable the pathogen to invade the host cells, were identified. Such a factor is the elevated levels of free iron in serum supporting the growth of the fungus and assisting in the spread at acidic pH in patients with diabetic ketoacidosis (Ibrahim et al., 2012). Patients receiving dialysis or blood transfusions are also highly susceptible to mucormycosis, the former is due to the treatment with the iron chelator deferoxamine, and the latter is because of the high amount of iron from the transfusions (Ibrahim et al., 2012).

Glucose starvation induces the overexpression of the receptor GRP78 (glucose-regulated protein), another virulence factor that facilitates the colonization and damage of endothelial cells (Ibrahim et al., 2012). Bruno et al. (2021) identified species-specific transcriptional antifungal host response pathways during the infection by *R. oryzae*. They found that the overexpression of *GRP78* is connected with the reduced glycolysis and down-regulation of glycolysis genes, which negatively affects the symptoms of patients who have diabetes.

A protein synthesis inhibitor, ricin-like toxin, mucoricin was described as a critical virulence factor for the pathogenesis of mucoralean fungi. This toxin has an N-glycosylase activity, similar to the plant-originated ricin, and inhibiting the protein prevents human cell damage *in vitro* (Soliman et al., 2021). The CotH, spore surface proteins initiate the infection by interacting with the host endothelial cells, and the copy number influences the virulence of the species. The higher copy number is associated with higher aggressiveness of the mucoralean species; in the absence of these proteins, the species is avirulent (Tahiri et al., 2023).

#### Cryptic species and taxonomy

*Rhizopus arrhizus* is a complex of closely related heterothallic species which can be cosmopolitan saprotrophs and pathogens of different organisms (Ghosh and Ray, 2011). Multilocus phylogenetic studies identified two species with outcrossing population structure in this coplex (Gryganskyi et al., 2010). The two varieties, *Rhizopus* var. *arrhizus* and var. *delemar*, form two well-supported clades, but there are no differences in ecology, geographic distribution, and pathogenicity (Dolatabadi et al., 2014). Additionally, the morphological differences between the varieties were slight and quantitative (Zheng et al., 2007). The genome and transcriptome analysis of 30 mucormycosis-causing fungi (including *Rhizopus* and *Mucor* species) underlined that there are no alterations in virulence and in the ability of these strains to cause diseases in humans (Chibucos et al., 2016).

#### Presence in the environment

*Rhizopus arrhizus* is one of the most common environmental species of the genus, and it has been found in different locations (e.g., grasslands, forests; Ribes et al., 2000) and different sources, like diseased plant materials (e.g., fava bean; Haddoudi et al., 2021). Infection by *Rhizopus* species can happen through inhalation of spores or ingesting contaminated food (Ribes et al., 2000). In one case study, a liver transplant recipient was shown to have contracted the pathogen by inhaling spores from compost during gardening (Munckhof et al., 1993).

*Rhizopus arrhizus* is a well-known but not severe plant pathogen. It causes, among others, root rot of fava bean (Haddoudi et al., 2021) and mulberry (Gnanesh et al., 2021), soft rot of garlic (Zhang et al., 2022), and head rot of sunflower (Zhou et al., 2018). This fungus can also be associated with the postharvest rot of several species, like apple (Khokhar et al., 2019), strawberry (Wang et al., 2015), and mandarin (Moosa et al., 2020).

Recently the prevalence of human-pathogenic species of Mucorales has been assessed in commercially available foodstuffs in France. Based on nrDNA Internal Transcribed Spacer (ITS) sequences, molecular identification revealed that *R. arrhizus* var. *arrhizus*, and *R. arrhizus* var. *delemar* could frequently be isolated from food products, like spices, herbs, herbal tea, and cereals (Mousavi et al., 2019). This draws attention to earlier findings that ingesting contaminated food can cause gastrointestinal mucormycoses, affecting mainly malnourished infants and children (Richardson, 2009).

#### Azole fungicide resistance

Voriconazole and fluconazole, two of the main antifungal chemicals, so-called short-tailed azoles, have no activity against Mucorales *in vitro* (Almyroudis et al., 2007) nor *in vivo* (Pagano et al., 2013; Shih et al., 2022). Thus, the recommended first line treatment of mucormycosis is AMB and POS, the long-tailed azoles (Dannaoui et al., 2003). This selective resistance to antifungals was proven by several studies presenting that POS, ITC, and ravuconazole showed good or low activity, but VRC does not affect mucormycosis causing fungi (Alastruey-Izquierdo et al., 2009; Vitale et al., 2012; Borman et al., 2021; Pfaller et al., 2021; Shih et al., 2022).

Cornely et al. (2019) published the global guideline on mucormycosis management considering the regional differences of the world. The recommended first-line treatment is the high-dose liposomal AMB and as salvage treatments, intravenous isavuconazole (ISA) and intravenous or delayed release tablet POS are suggested. This guideline underscores the importance of early diagnosis and the recognition of disease patterns.

Osaigbovo et al. (2023) reviewed mucormycosis cases from Africa from 1960 until 2022, discussing the treatments and overall survival rates. Approximately 82% of the patients received antifungal therapy, in 82.3% of these cases, AMB was used. In 10% of cases, POS was used for the treatment, while VRC and FLC, which were generally considered ineffective, were applied in 5.4 and 2.4% of the cases, respectively. Sixty percent of the patients survived, and ~40% died (Osaigbovo et al., 2023). Probably, the difficult accessibility explains the low use of antifungal agents, as it was presented that in Africa liposomal AMB was available in < 20% and POS in 5% of the institutions (Osaigbovo et al., 2023).

The invasive fungal infection data was summarized, including mucormycosis, from the SENTRY Antifungal Surveillance Program, the longest-running antimicrobial surveillance programs monitoring the resistance patterns of pathogens (Pfaller et al., 2021, 2022). Pfaller et al. (2021) compared the SENTRY data with the susceptibility data of several strains tested with three azoles (VRC, POS, and ISA). They confirmed that VRC is ineffective against mucoralean species, POS was superior (MICs ranging from 0.5 to 4 mg) and the MIC values in the case of ISA were as high as with VRC when *R. oryzae* was tested (Pfaller et al., 2021). When younger (between 18 and 64 years of age) and older age group was compared based on the distribution of opportunistic invasive fungal infections and antifungal susceptibility data, they found that resistance to triazoles was less common in the older population (Pfaller et al., 2022). The antifungal susceptibility data were consistent with earlier results where POS was the most effective, and all the mucoralean species were resistant to VRC (Pfaller et al., 2022). Analyzation of nationwide, population-based data in Taiwan revealed that treatment with POS was associated with lower in-hospital mortality and higher survival probability at 90 days after discharge compared to VRC (Shih et al., 2022). A review of clinical combination therapies against Mucorales showed that some synergistic interactions can be achieved (e.g., echinocandins with either azoles or AMB) and never antagonism. In some cases, 50% synergism was detected when AMB was combined with POS (Schwarz et al., 2019).

Only scarce information is available on the molecular basis of the antifungal resistance mechanism in Mucoralean fungi. Chau et al. (2006) found that two amino acid changes, Y132F and F145M (Figure 1B), substitutions from tyrosine to phenylalanine and phenylalanine to methionine, respectively, impact susceptibility to fluconazole without affecting either posaconazole or itraconazole. Genome sequence analysis of *R. oryzae*, isolated from a fatal case of mucormycosis, revealed evidence of an ancestral whole-genome duplication and recent gene duplications. This genome expansion affected the genes involved in the cell wall, cell membrane and ergosterol biosynthetic pathway, including *ERG11*, coding the principal target of azole drugs (Ma et al., 2009). The increased number of copies could contribute to fungicide resistance (Figure 1B), as was shown in plant pathogenic fungi (Jones et al., 2014).

It has been repeatedly shown that the naturally occurring Y129F and the *CYP51* gene are exclusively responsible for these susceptibility patterns (Caramalho et al., 2017; Macedo et al., 2018, 2021). Comparison of the paralogous pair *CYP* genes (*CYP51 F1* and *CYP51 F5*) in different *Rhizopus* and *Mucor* species revealed that the Y129F mutation is conserved in all Mucorales. This substitution may change the structure of the protein resulting in the resistance to short-tailed azoles but it has no impact on binding the long-tailed azoles, like posaconazole (Caramalho et al., 2017).

#### Diagnosis of *R. arrhizus* and fungicide resistance

Identification of Mucorales species can be made with antibody-based techniques, like immunoelectrophoresis, Enzyme-Linked Immunosorbent Assay (ELISA), and immunodiffusion assays, or with visualization using computerized tomography (CT) scan or positron emission tomography-computed tomography (PET/CT; Ribes et al., 2000) or with detecting Mucorales-specific T cells (Potenza et al., 2016). These methods can be used effectively in the more advanced stages of infection. For more specificity and the rapid diagnosis of the causal agent of mucormycosis, the PCR-based approaches have more potential. For the DNA-based identification Restriction Fragment Length Polymorphism (RFLP; Machouart et al., 2006), semi-nested PCR (Hammond et al., 2011), sequencing of the ITS region (Keisling et al., 2014), and qPCR (Scherer et al., 2018) are the frequently utilized methods. Besides the ITS region, the mucoralean-specific spore coating protein homolog gene, CotH, can be the target for PCR amplification and identification of *R. arrhizus* and other mucoralean species (Baldin et al., 2018). A commercially distributed, multiplex qPCR assay, MucorGenius (Pathonostics), can detect clinically relevant species.

The reproducibility and performance of different Mucorales qPCR assays were evaluated in an interlaboratory collaboration recently (Rocchi et al., 2021). The variability between laboratories was minimal and the concordance was excellent, only the observed Cq values varied between different qPCR platforms. Overall the use of qPCR based technics is highly recommended and encouraged for detecting Mucorales DNA, especially from serum (Rocchi et al., 2021). Mendonça et al. (2022) developed a qPCR multiplex method for detecting *R. arrhizus* and four *Aspergillus* species. The qPCR is based on SYBR Green and a melting curve analysis. It enables the simultaneous diagnosis of these invasive filamentous fungi either from culture or plasma, even when a small amount of DNA is available (Mendonça et al., 2022).

The detection methods of mucormycoses, including histopathology and direct microscopy were discussed in detail by Skiada et al. (2020).

For the evaluation of the susceptibility for the antifungal drugs Clinical and Laboratory Standards Institute (CLSI) and European Committee on Antimicrobial Susceptibility Testing (EUCAST) reference methods are currently available and recommended (Cornely et al., 2014; Dannaoui, 2017). Caramalho et al. (2015) compared the efficacy of these broth microdilution assays, CLSI and EUCAST, with the commercially available Etest^®^. The former test is easy-to-use and time efficient but they found low levels of agreement of the results gained with Etest^®^ and EUCAST for Mucorales (Caramalho et al., 2015).

Utilizing the automated BioCell-Tracer^®^ (BCT) system enables the assessment of the effect of different antifungals in different concentrations directly on growing hyphae, even in the emergence stage (Taguchi et al., 1995; Fonseca et al., 2018). Fonseca et al. (2018) found that hyphae are more susceptible than conidia to AMB, itraconazole, and terbinafine based on the patterns using the BCT system.

### *Fusarium solani* species complex

#### A complex of polyphagous pathogens and cryptic species

The *F. solani* species complex (FSSC) consists of several plant pathogens, endophytes, decomposers, symbionts of insects, and opportunistic pathogens of humans and animals (Schroers et al., 2016). FSSC is a phylogenetically and biologically complex group that is responsible for approximately two-thirds of all fusarioses cases worldwide (O'Donnell et al., 2008; Guarro, 2013; Batista et al., 2020; Bupha-Intr et al., 2021). Fusarioses are relatively rare compared to invasive candidiasis and aspergillosis, but FSSC can cause life-threatening opportunistic infections in immunocompromised and immunosuppressed patients (O'Donnell et al., 2020; Bupha-Intr et al., 2021). Invasive fusarioses, including bloodstream infections, have a high mortality due to the widespread antifungal resistance and frequently delayed diagnosis (Rajabzadeh et al., 2020; Huang et al., 2023). Nevertheless, FSSC can cause disease either on superficial or traumatized tissues in immunocompetent individuals, like keratitis, often associated with improper contact lens wear (Chang et al., 2006; Niu et al., 2020) and eumycetoma in patients with diabetes mellitus (Das et al., 2021).

#### Pathogenesis and virulence factors

The most common route of infection is through the respiratory tract, but could happen also by paronychia infection (onychomycosis) and trauma of the skin and eyes (keratitis; Batista et al., 2020; Bupha-Intr et al., 2021). The gastrointestinal tract represents an unlikely but slightly possible gateway to infection i.e., the consumption of food contaminated by *Fusarium* propagules (Nelson et al., 1994). Skin or nail infection can be the secondary source of inoculum prior to invasive infection, which can involve the bloodstream, bones, joints, etc. and more attention should be paid to it during physical investigations of the patients (Batista et al., 2020). The central nervous system can rarely be involved, e.g., eye pain, headache, and focal neurologic deficits (Vadhan et al., 2022).

*Fusarium* species are very effective pathogens due to the capability of dispersing long distances, sporulation in tissue and blood, production of mycotoxins and enzymes, and intrinsic multi-drug resistance (Bupha-Intr et al., 2021). Mycotoxins, like trichothecenes, inhibit humoral and cellular immunity, and protease enzymes produced by *F. solani* can cause corneal ulceration and destroy the epithelial cells in keratitis (Niu et al., 2020). Contact lens wearing is one of the risk factors and source of infection for fungal keratitis because *Fusarium* species can colonize the interior matrix of the lens and degrade the hydrophilic polymer components (Nelson et al., 1994).

*Fusarium solani* species due to keratinolytic activity are able to invade healthy nails and develop onychomycosis (Khan et al., 2019). In the infected nail tissue, through the adventitious sporulation, yeast-like, thick-walled budding structures are formed (Khan et al., 2019). This also illustrates that the morphology of the pathogen can alter depending on the invaded tissue, which should be considered during the identification of the pathogen (Sokolova et al., 2022).

#### Taxonomy

The name *F. solani* is widely used in plant pathology and clinics. Phylogenetic analyses have repeatedly shown that this taxon represents a complex group of cryptic species (Schroers et al., 2016). The *F. solani* species complex (FSSC) consists of three major evolutionary subgroups, and all clinically relevant species belong to one of those clades (O'Donnell et al., 2008; Geiser et al., 2021). Recently two main concepts conflicted with the phylogenetic position of *F. solani* species. Sandoval-Denis and Crous (2018) stated that the genus *Fusarium* is polyphyletic, and many clades are artificially arranged into it. They have suggested a new taxonomic approach based on four loci (nDNA ITS; elongation factor-1 alpha, EF-1α, nuclear Large Subunit rDNA, LSU; and RNA polymerase II 2nd largest subunit, RPB2) and morphological characters and introduced the generic name *Neocosmospora* for the FSSC complex, including several medically important taxa (Sandoval-Denis and Crous, 2018). On the contrary, O'Donnell et al. (2020) argued that FSSC had to be considered as belonging to the genus *Fusarium* and emphasized that splitting medically important FSSC species into different genera is unnecessary and misleading. They aware clinicians about the importance of proper identification because *Fusarium* species show a high level of resistance to several antifungals, and misidentification can cause fatal outcomes (O'Donnell et al., 2020).

Geiser et al. (2021) presented a robust phylogeny based on 19 protein-coding genes derived from whole-genome sequences. The results supported the monophyly of *Fusarium*, including the FSSC. The FSSC group was further examined based on 3-locus (RPB2, EF-1α, and nDNA ITS) phylogeny and the position within the genus *Fusarium* was confirmed (Geiser et al., 2021).

#### Prevalence of FSSC in the environment

The main source of inocula infecting humans are contaminated plant materials, soil, inhalation of polluted air, and as it was mentioned earlier, contaminated contact lens (Al-Hatmi et al., 2016). Short et al. (2011) found that hospital plumbing systems are also dangerous reservoirs for *Fusarium* inoculum. Comparing the diversity of clinical and plumbing drain-associated isolates revealed that *Fusarium* samples sharing the identical multilocus haplotypes are widespread and can be frequently isolated from both sources (Short et al., 2011). The results of another extensive survey of medical institutions also highlighted that the sewerage system and drain outlets can host *Fusarium* inoculum and droplet-mediated spread is a potential source of invasive fusarioses (Hino et al., 2020).

It has been repeatedly shown that FSSC strains isolated from plant materials are closely related to clinical species. Based on phylogenetic analysis, these strains group into the same clade as species causing human infection. For example, FSSC was responsible for crown rot and stem canker of pistachio (Crespo et al., 2019), wilt and root rot in both watermelon and melon crops (González et al., 2020), and citrus dry root rot on sour orange rootstock (Kunta et al., 2015). In addition, one of the causal agent of human fusarioses, *Neocosmospora keratoplastica* (*F. keratoplasticum*), member of the FSSC, was described for the first time as responsible for wilting and root rotting of Cucurbitaceae (González et al., 2020). They showed that this species is also widespread in agricultural areas, and most likely, its prevalence is underestimated.

Homa et al. (2018) studied clinical (from corneal scrapings) and environmental (from roots and soil) FSSC isolates from South India. The virulence of the strains was compared using *Drosophila melanogaster* model and they found that the pathogenicity was independent from the origin of the isolates.

James et al. (2022) analyzed 15 clinical (collected from nail, skin, corneal scrapings, and blood) and 15 environmental (including plant materials) FSSC isolates based on TEF1-a (translation elongation factor alpha, also known as EF1-α) and RPB2 Sequences. Three of the five clinical species examined in this work were also isolated from plants among other materials, e.g., *F. keratoplasticum* was isolated from nails, skin, and grass; *F. falciforme* was isolated from eyes, soil, tobacco, honeydew and straw compost. Emphasizing even further the possible environmental and agricultural origin of the clinically important species (James et al., 2022).

Mehl and Epstein (2007) were the first to prove the plant pathogenicity of human clinical FSSC isolates. During pathogenicity tests, zucchini fruits were inoculated with FSSC strains isolated from postharvest cucurbit fruits, human clinical, sewage, and hospital environment sources. There were no significant differences in pathogenicity between isolates from different sources. They proved the identity of the clinical and plant pathogenic isolates based on the multilocus phylogeny and sexual compatibility (Mehl and Epstein, 2007).

Meza-Menchaca et al. (2020) provided more details about the plant pathogenic characteristics of clinical *Fusarium* strains originating from keratitis samples. They performed *in vitro* assays with detached leaves, seedlings, and small pieces of nails as a human onychomycosis assay. In all experiments, *Fusarium* conidia germinated, colonized the plant and human tissues, and in the case of *in vitro* seedlings, deterred the growth. These *in vitro* experiments showed that *F. solani* strains, the causative agents of keratitis, are able to reinfect plants and humans (Meza-Menchaca et al., 2020).

#### Azole antifungal susceptibility and resistance mechanisms

The mortality among patients suffering from fusariosis is undoubtedly high because of the resistance (both intrinsic and secondary resistance) to several antifungals, especially, if the central nervous system is involved (Garcia et al., 2015). Fusarial species show different levels of resistance to different antifungals, e.g., resistance to FLC and ITC is widespread, while VRC and POS show some activity (Al-Hatmi et al., 2016). Often it is the combination of different antifungals that makes the treatment effective, like a combination of VRC with AMB (Ruíz-Cendoya et al., 2008) or with micafungin (Heyn et al., 2005) or natamycin with terbinafine (Homa et al., 2013).

The recommended antifungal treatment for invasive fusarioses as a first-line therapy is lipid formulation of AMB or VRC (Bupha-Intr et al., 2021). The combination therapy with VRC and AMB is moderately recommended by Bupha-Intr et al. (2021). Salvage therapy with POS is also moderately recommended and only when the isolate's susceptibility is determined, and the suitable formulation is available. In the case of endophthalmitis caused by *Fusarium* species, systemic and intravitreal antifungal agents are needed. Since VRC has a good capability to penetrate the eye, the use of VRC is recommended over AMB (Bupha-Intr et al., 2021).

Until recently, the exact mechanisms of resistance were not fully understood. The overinduction the *CYP51A* gene (Figure 1B) was observed after exposure to VRC and other azoles, including the agricultural fungicide, tebuconazole (D'agostino et al., 2018). James et al. (2021) showed that ABC1 ortholog transporters (Figure 1B) contribute to the azole resistance phenotypes in *F. keratoplasticum* and *F. petroliphilum*, the two most frequently isolated members of FSSC in the clinic. The same research group reported (James et al., 2020) a 23 bp length deletion in the *CYP51A* promoter accompanied by high VRC MIC values. They studied isolates from clinical and environmental samples, the former originated from nail, skin, corneal scraping, and blood, and the latter were collected from soil and plant debris. The existence of promoter deletion and VRC resistance were both found either in clinical or environmental samples. They suggested that this promoter deletion can be used as a molecular marker of VRC resistance (James et al., 2020).

Recently, the possible role of SNPs were studied investigating the mutations in Cyp51 protein sequences in clinical and environmental FSSC strains (Vermeulen et al., 2022). The susceptibility to azoles and MIC values of these strains were determined using the CLSI method. The MIC values were high for all antifungals for all strains but presented a broad range for VRC and isavuconazole. Comparing the protein sequences with the sequences of resistant *Aspergillus fumigatus* strains revealed that the mutations at positions 170, 218, 253, and 422 decrease the susceptibility of these FSSC fungi to azoles. These amino acid changes might influence the protein structure, ligand binding, and locking of the protein against the membrane (Vermeulen et al., 2022).

Another possible mode of fungicide resistance in FSSC, without alteration of the Cyp51 protein, is biofilm formation. Córdova-Alcántara et al. (2019) define biofilms as a complex of surface-associated microbial populations surrounded by a protective extracellular matrix (Córdova-Alcántara et al., 2019). FSSC biofilm can increase the tolerance to ultraviolet light and decrease the susceptibility to different antifungals, including VRC and AMB. The matrix of biofilms serve as physical barrier against chemical and biological substances and influences the activation of efflux pumps and the sterol content of the membrane (Córdova-Alcántara et al., 2019).

#### Diagnostics of FSSC and fungicide resistance

As with other species, accurate species identification is crucial for the selection of appropriate treatments (Al-Hatmi et al., 2017a). The sampling procedure is also critical because coinfection of fusarial species is possible; thus, examination of single strains should not be enough (Guarro, 2013).

*In vivo* confocal microscopy is an important diagnostic tool for fungal keratitis, which can be used to examine hyphae directly in the cornea (Niu et al., 2020). This method enables the observation and early detection of depth fungal infection and allows for starting appropriate treatment in time (Niu et al., 2020; Sourlis et al., 2022). Besides of the outstanding utility of confocal microscopy, it needs an expensive tool and an experienced user for the operation (Niu et al., 2020).

Identifying the species based on morphological features is challenging because of the overlapping characteristics of different species and members of the same complex (Al-Hatmi et al., 2017a). Therefore, proteomic-, or DNA-based methods should be involved for the reproducibility and reliability. The matrix-assisted laser desorption ionization–time-of-flight (MALDI–TOF) analysis, a proteomic-based strategy, is a mass spectral analysis using a database for the identification. The method gives results within 1 h but has the disadvantage that it cannot identify species that are missing from the database (Marinach-Patrice et al., 2009).

Homa et al. (2013) developed a FSSC-specific molecular tool for rapid identification. They found a recognition site of EcoRI enzyme in the *EF-1*α sequences of members of FSSC. The enzymatic digestion of the PCR products of *EF-1*α resulted two of ca. 480 and 270 bp long fragments. This pattern was specific for all isolates belonging to FSSC. O'Donnell et al. (2007) developed another rapid method for the identification, a single-well diagnostic assay using flow cytometry and fluorescent microsphere technology. Allele-specific probes were designed based on *RPB2* sequences, which allow the simultaneous identification of human pathogenic species and species complexes (O'Donnell et al., 2007).

Sequencing different loci is one of the most widespread and most effective identification methods. The universal fungal barcode, ITS region (Schoch et al., 2012) usually does not give enough information to distinguish *Fusarium* species. For the thorough identification the elongation factor, RNA polymerase II and beta-tubulin gene sequences are recommended (O'Donnell et al., 2013, 2015).

A broad range of *Fusarium* species can be detected with a probe-based real-time PCR assay. Springer et al. (2019) designed a 28S-based analysis utilizing a specific hydrolysis probe which can be applied for the diagnosis of fusarioses from bronchoalveolar lavage fluid samples. Another PCR-based method employing a special instrument with electrochemical detection system was developed by Zhang et al. (2020) for the identification of 15 fungal pathogens directly from blood cultures. The GenMark Dx ePlex instrument is able to perform all steps from processing the blood samples until the detection of the target DNA (Zhang et al., 2020). This method is suitable for simultaneous analysis of large numbers of samples, easy-to-use, and answers are obtained in a short time (Zhang et al., 2020), although a particular instrument is needed.

To detect fungicid-resistance of FSSC isolates, generally broth microdilution methods are applied (Al-Hatmi et al., 2017b). For a detailed review of susceptibility testing methods applied for *Fusarium* species, see Al-Hatmi et al. (2017a). To our knowledge, very few DNA-based molecular techniques are available, and only Sanger sequencing of CYP51 gene and its promoter region is used (James et al., 2020).

Mainly CLSI and EUCAST methods and commercially available rapid Etest^®^ method are utilized for the antifungal susceptibility testing of FSSC; and all provide MIC data and are sufficient for optimizing the therapy of individual patients (e.g., Arendrup et al., 2020). However in some cases, a poor correlation was found between the *in vitro* susceptibility data and the outcome of the patient's treatment (Al-Hatmi et al., 2017b). In the case of eumycetoma, the FSSC isolates showed high MIC values for several antifungals, like ITC. However, the patients responded well to ITC and recovered from the infection (Al-Hatmi et al., 2017b).

In an MT2 microplate-based assay, the fungal mycelium is suspended in inoculating fluid, and then the MT2 microplate wells are filled with suspension (Frac et al., 2016). The method was tested on clinically and agriculturally (e.g., tebuconazol) used antifungals. Compared to the whole-plate approach, this process is more simple and cost-effective (Frac et al., 2016).

Blaize et al. (2021) determined the antifungal susceptibility of 182 *Fusarium* isolates to AMB, VRC, POS, ISA, and terbinafine. They used EUCAST standardized methodology for all isolates and gradient concentration strip methods for 77 isolates to test the correlation between the two methods. They found that the correlation changed with the time of growth, e.g., in the case of POS a good correlation was obtained within first 24 h. However, for AMB the best correlation was gained after 72 h of growth. Overall the MIC values gained with the gradient strip method were lower than the EUCAST method. Furthermore, for voriconazole, the correlation coefficients were lower regardless of the growth period (Blaize et al., 2021).

A malignant keratitis case caused by a highly-resistant *F. solani* strain highlights the importance of proper detection, identification, and antifungal susceptibility testing (Schrecker et al., 2021). The causal agent was identified by morphology, ITS and *TEF* sequences as *F. tonkinense* belonging to the *F. solani* species complex. The isolate showed high MIC values for several antifungals except for natamycin. The treatment included topical natamycin and polyhexamethylene biguanide, systemic VRC, terbinafine, and intravitreal injections of VRC and AMB. The patient underwent two keratoplasty, and at the end, the vitreous body also became affected, and the endophthalmitis was rapidly progressing. After several months of treatment failure, the eye was enucleated (Schrecker et al., 2021). Nevertheless, the failure could be explained by the biofilm formation by the fungus. The hyphae adhere to laminins, fibronectins and collagens on the cornea and create the biofilm that protects the colony from antifungals. When the removed eye was examined fungal elements were not detectable. The authors hypothesized that the antifungal treatment finally eliminated the causative agent, and the immune response overreaction resulted in acute necrotizing inflammation (Schrecker et al., 2021).

Bloodstream infection represents minority, only the 1% of all *Fusarium* infections (Thomas et al., 2020), although the high mortality highlights its importance (Rajabzadeh et al., 2020; Huang et al., 2023). Especially in immunocompromised patients, a broad-spectrum antifungal agent and combination therapy with systemic antifungals are recommended (Rajabzadeh et al., 2020; Huang et al., 2023).

###  *Curvularia lunata*

#### A neglected trans-kingdom pathogen

Eumycetoma was declared as a neglected tropical disease by the WHO, present mainly in the tropical and subtropical regions (known also as the “Mycetoma Belt”) and sporadically found in other countries (Zijlstra et al., 2016). Several species are proven as agents of eumycetoma, which are now included in the high priority group on the FPPL. This granulomatous infection affects mainly the skin and subcutaneous tissue, sometimes with bone involvement (Agarwal et al., 2021). Usually, the lower extremities are involved; patients are typically farmers and people working barefoot on agricultural fields (Zijlstra et al., 2016). Presumably, the gateway of infection is a traumatic injury reaching subcutaneous layers, like a thorn prick (Santona et al., 2021) or the respiratory system (Skovrlj et al., 2014).

*Curvularia lunata* is one of the eumycetoma causative agents infecting mammals and plants. Besides eumycetoma, it can cause several mycotic diseases like rhinosinusitis with orbital cellulitis (Narula et al., 2020) and keratitis (Niu et al., 2020), and it is one of the most frequent species involved in local and invasive phaeohyphomycoses (Revankar et al., 2017). It is a keratinophilic species, producing keratinases that enable the fungus to colonize the skin (Wang et al., 2023). As other dematiaceous fungi, there is melanin, a well-known virulence factor, in the cell wall of *C*. *lunata* to avoid the host immune system (Skovrlj et al., 2014).

As a plant pathogen, *C. lunata* causes early blight disease of tomato (Abdelfatah et al., 2021), leaf spot of corn (Manzar et al., 2022), postharvest fruit rot of banana (Khan and Javaid, 2020), rice seedling rot disease (Limtong et al., 2020), and foliar necrotic spots of Andropogon grass (Santos et al., 2018) and *Brassica rapa* (Wonglom et al., 2018).

#### Identification and diagnosis

Identification of plant pathogen *C. lunata* strains is based mainly on ITS (Abdelfatah et al., 2021), Ras-related (Clg2p) gene (Hou et al., 2013), TEF1 and glyceraldehyde 3-phosphate dehydrogenase sequences (Garcia-Aroca et al., 2018). Culture characteristics, morphology and conidiation pattern are also frequently used for identification in plant pathology and clinics (Dubey et al., 2019; Khan and Javaid, 2020), and a next generation sequencing based approach has been published recently (Santona et al., 2022). Detailed information on detection of eumycetoma causing agents were published recently (Hao et al., 2022; Husain et al., 2022).

#### Treatment and antifungal susceptibility

Limited knowledge is available about the susceptibility and resistance of eumycetoma causing agents, and the lack of available CLSI criteria for *C. lunata* often leads to delayed and prolonged treatment (Dubey et al., 2019; Al-Odaini et al., 2022). The primary management of eumycetoma are surgical debridement along with systemic antifungals (Agarwal et al., 2021).

Different antifungal agents have very diverse effects on *C. lunata*. Based on checkerboard broth microdilution method VRC was the most effective, while terbinafine, AMB, ITC and ketoconazole had moderate activity, and FLC was the least efficient antifungal agent (Al-Odaini et al., 2022). Agarwal et al. (2021) reported that terbinafine is effective only in combination with ITC; VRC and POS have both good *in vitro* activity, while echinocandins and AMB have poor *in vitro* outcome. In an other *in vitro* study AMP, terbinafine and POS were the superior antifungals, the other azoles had only moderate *in vitro* activity against *C. lunata* (Revankar et al., 2017).

Depending on the method used for the evaluation of the *in vitro* activity of antifungals, different outcome on effectiveness was learned. Zheng et al. (2019) compared the susceptibility of dematiaceous fungi to nine antifungal agents using Sensititre YeastOne^®^ colorimetric antifungal panels and the CLSI M38-A2 reference broth microdilution method. For most common antifungals (e.g., AMB, VRC, ITC, FLC, and POS) the agreement between the two methods was high, while for echinocandins (e.g., caspofungin) was very variable among different species, and for *C. lunata* there was a 60-fold difference between the MIC values. They concluded that the YeastOne^®^ method has the advantages of simplicity and shortening the test time, but the color changing, which indicates the MIC values, was ambiguous if echinocandins are tested. The same research group (Zheng et al., 2020) tested azoles on 84 clinical isolates of dematiaceous fungi, including *C. lunata*, and they found that ravuconazole and ISA are the superior antifungals for inhibition of the fungal infection.

Only AMB was given as antifungal treatment to a 69-year-old male heart transplant recipient who had painful skin lesions, multiple deep browns, cutaneous nodules and nodes, and the lesions disseminated to the whole arm (Tessari et al., 2003). One week before the first symptoms he had got a splinter during gardening. Hyperchromic maculopapular skin lesions spread on the whole skin surface and to the oral mucosa soon after the first antifungal treatment. The causal agent was identified as *C. lunata*. The infection got worse: a disseminated phaeohyphomycosis developed involving the skin, mouth, esophagus and the upper lobe of the right lung. The patient's condition worsened rapidly, and he passed away in a few days. The massive immunosuppression with strong drugs and delay in the diagnosis contributed to the fatal outcome of this case (Tessari et al., 2003).

A 66-year-old diabetic patient performed a rapidly growing lesion on her face soon after harvesting corn (Wang et al., 2023). Interestingly she was not treated with antifungal agents only with surgery combined with photodynamic therapy and 5-aminolevulinic acid. The patient completely recovered within a short time (Wang et al., 2023).

An immunocompetent but heavy cannabis- and alcohol-consuming 33-year-old patient was diagnosed with *Curvularia* infection in his brainstem. He suffered from headaches, nausea, vomiting, and a recent weight loss (Skovrlj et al., 2014). First, a lesion was found with MRI within the right medulla, and then a CT scan revealed a lesion in the left lung. During an open biopsy, an encapsulated lesion was found tightened to the brainstem. The microbiological examination revealed the *Curvularia* infection and an antifungal treatment with voriconazole, flucytosine, and liposomal amphotericin B was started. Despite treatment, the patient's condition worsened and he needed another surgery. After the surgical debridement and treatment with voriconazole, the patient recovered from the infection (Skovrlj et al., 2014).

As far, we know there is no information on the antifungal resistance mechanisms in *C. lunata*.

## Conclusions and future remarks

Resistance to azoles is an increasingly serious problem in human medicine and agriculture, where azoles are the first-line fungicides of defense against pathogens (Gisi, 2014). Thus, the proper and accurate identification of the causal agents and detection of resistance are crucial in both fields. The incorrect identification can mislead the treatment in medicine and agriculture, i.e., delay patients' recovery, and it contributes to the spread of resistant pathogens and loss of efficacy of fungicides. Special attention should be given to the relevance of the resistant fungal pathogens in the environment, therefore in agriculture and forestry (e.g., wood preservation) the widespread usage of azoles enhance the abundance of resistant fungal populations. Since these resistant populations pose a threat in the human clinic, proper identification is undoubtedly necessary. Therefore, the development and improvement of molecular methods for identification and diagnostics are increasingly urgent.

For future developments, promising methods can be e.g., LAMP and digital droplet PCR (ddPCR) based techniques. The former is rapid and low-cost, with great potential for early diagnosis (Yu et al., 2019). The ddPCR is also a quick method with high selectivity and the ability to quantify the pathogens (Lei et al., 2021).

Developing new and improved antifungal agents can enhance the outcome of human therapy (Mota Fernandes et al., 2021). Novel chemicals and improved azoles are around the corner, like olorofilm, which inhibits the pyrimidine biosynthesis in fungi and alters DNA synthesis, cell growth and division (Georgacopoulos et al., 2021). While luliconazole is a well-known antifungal, the interest toward it has been emerging recently. It is highly lipophilic, but proper nanocarriers can enhance its solubility and permeability that improve the therapeutic effect. In addition, luliconazole has a broad-spectrum antifungal activity making this fungicide a promising future antifungal agent in clinics (Dos Santos Porto et al., 2021).

We believe that the development of molecular methods is a crucial issue, because classical methods, like broth microdilution, may not be enough in all cases and should be complemented by other, DNA-based approaches. There is a need for methods to be used routinely, which are rapid, cost-effective and high-throughput if necessary.

An open question is whether fungi, which are virulent on both plants and humans, and become azole resistant in the clinics are able to infect plant material when released into the environment (Figure 1). Similarly, it is not known, whether there is any kind of transmission of fungal pathogens from human to plants, plant materials. It is generally considered as a one-way process, or only one direction is considered, studying the human infection. However, we should bear in mind, that these fungi are capable for the opposite way of the transmission, nevertheless, these cases might be sporadic compared to those when fungi with agricultural and environmental origin cause human infections. Since resistance to different antifungals is a continously emerging threat in all fields and multidisciplinar approach and collaboration is required across the One Health spectrum (World Health Organization, 2022).

## Author contributions

AP: Conceptualization, Funding acquisition, Writing—original draft. RB: Writing—review & editing. GK: Conceptualization, Writing—review & editing.
